# Integrated Spatial Modulation and STBC-VBLAST Design toward Efficient MIMO Transmission

**DOI:** 10.3390/s22134719

**Published:** 2022-06-22

**Authors:** Kaiyuan Huang, Yue Xiao, Lizhe Liu, Yong Li, Zhiqun Song, Bin Wang, Xingjian Li

**Affiliations:** 1 The Key Laboratory of Science and Technology on Communications, University of Electronic Science and Technology of China, Chengdu 611731, China; kaiyuanhuang12138@163.com; 2 Science and Technology on Communication Networks Laboratory, Shijiazhuang 050081, China; liu_lizhe@sina.com (L.L.); young_li_54@126.com (Y.L.); zhiqunsong@163.com (Z.S.); 18705442162@163.com (B.W.); lixingjianjan@163.com (X.L.)

**Keywords:** vertical Bell-labs layered space-time (VBLAST), space-time block code (STBC), spatial modulation (SM), transmit power allocation (TPA)

## Abstract

In this contribution, the concept of spatial modulation (SM) is firstly integrated into the structure of space-time block codes (STBC)-aided vertical Bell-labs layered space-time (VBLAST) systems, in order to strike a balanced tradeoff among bit error ratio (BER), spectral efficiency and computational complexity. First of all, in order to enhance the BER performance of STBC-VBLAST, we advocate an effective transmit power allocation (TPA) scheme with negligible implementation costs, while dividing the STBC and VBLAST layers with alleviated interference, so as to facilitate combination with SM. Then, we further utilize the unique structure of SM for enhancing the spectral efficiency of original STBC-VBLAST, wherein the information is conveyed by not only the amplitude/phase modulation (APM) symbols but also the antenna indices. In addition, constellation sets of STBC symbols are specifically designed to be rotated to make full use of the degrees of freedom. Finally, the performance advantages of the above-mentioned structures over traditional STBC-VBLAST are demonstrated by the theoretical derivation of a closed-form expression for the union bound on the bit error probability for various spectral efficiencies, and they are supported by simulation results.

## 1. Introduction

Over the past two decades, multiple-input multiple-output (MIMO) systems, which are capable of providing increased capacity in fading environments, have attracted considerable attention. Toward the development of the sixth generation (6G) [1] in pursuit of even higher spectral efficiency and transmission rates, and high reliability, MIMO is still desired to be developed to meet the above challenges in different wireless scenarios through careful designs [2].

In the view of a traditional MIMO design, vertical Bell-labs layered space-time (VBLAST) [3,4] and space-time block coding (STBC) [5,6] have been favored to improve the performance of MIMO systems. On the one hand, in VBLAST systems, each data stream is independently transmitted in a certain antenna at the same time, and thus high spectral efficiency occurs with a high level of inter-channel interference (ICI) at the receiver. Furthermore, since the complexity of the optimal detector for VBLAST systems grows exponentially, some low-complexity linear decoders, such as zero-forcing (ZF), minimum mean-squared error (MMSE) and QR decomposition, have been employed to degrade the error performance. On the other hand, STBC is an attractive technique that can provide transmit diversity, mitigate the effect of multipath fading and offer implementation simplicity and low decoding complexity. In particular, Alamouti’s space-time block coding is an important scheme for providing full transmission and receiving antenna diversity, and the maximum code rate for two transmit antennas; the maximum possible code rate is 3/4 for more than two antennas. Furthermore, to combine the advantages of both VBLAST and STBC, the joint design of STBC-VBLAST has been widely considered, as in [7,8,9,10,11,12]. The design goal of the above structure focuses on the attainable diversity provided by STBC toward enhanced resistance to the interference caused by VBLAST; however, the degradation of the spectral efficiency remains a huge challenge.

Meanwhile, as a class of new MIMO assisted modulation or coding techniques, spatial modulation (SM) [13,14,15] has attracted wide attention due to its special structure that extends two dimensional signal constellations to a third dimension, called the spatial (antenna) dimension, so as to convey the information by both the amplitude/phase modulation (APM) techniques and the antenna indices, while offering a low-cost implementation [16,17,18]. From a space-time coding point of view, SM provides enhanced spectral efficiency over traditional STBC and alleviated interference over VBLAST. Therefore, it has the potential to be integrated into the current MIMO structure to create more efficient designs [4,19,20]. However, until now, the introduction of SM into the design of STBC-VBLAST, in order to enhance the system’s performance with affordable complexity, remains an open challenge.

In a nutshell, of prime concern in this paper is firstly utilizing the unique structure of SM to enhance the original STBC-VBLAST system by improving its error performance and spectral efficiency. Meanwhile, we will consider transmitter power allocation (TPA) [21] as an efficient way to alleviate the interface in the developed STBC-VBLAST-SM structure. The main contributions of this paper can be summarized as follows:

- A new TPA scheme, intergrated into the original STBC-VBLAST structure, is proposed to improve the BER performance of STBC-VBLAST. A specific class of STBC-VBLAST systems with TPA, in which two of the antennas transmit Alamouti’s STBC and the others transmit independent data streams [22], was chosen as the target to exploit. Simulation results and performance analysis indicate that the TPA scheme can significantly enhance the BER performance of STBC-VBLAST systems in terms of maximum-likelihood (ML), MMSE and ZF with a negligible implementation cost.
- A new MIMO transmission scheme, named STBC-VBLAST-SM, is also presented, in which information is conveyed by STBC matrices, VBLAST symbols and the combinations of the antennas from which STBC matrices are transmitted. As a result, STBC-VBLAST-SM systems are able to obtain higher spectral efficiency than STBC-VBLAST systems. Similarly to TPA, the STBC-VBLAST-SM systems with Alamouti’s STBC were chosen as the target to exploit. Simultaneously, against binary phase-shift keying (BPSK) modulation, in order to make full use of the degrees of freedom, constellation sets of STBC layers are rotated to enhance the diversity. Computer simulations and theoretical analysis verified the necessity of the process of rotation.
- We also focus on theoretical work to demonstrate that the STBC-VBLAST systems with TPA and STBC-VBLAST-SM systems have advantages over the original STBC-VBLAST systems with an optimal decoder. A closed-form expression for the union bound on the bit error probability of the STBC-VBLAST with TPA and the STBC-VBLAST-SM scheme is also derived to support our results. The derived upper bound is shown to become very tight as the signal-to-noise ratio (SNR) increases.

The remainder of this paper is organized as follows. In Section 2, we introduce the STBC-VBLAST with TPA and STBC-VBLAST-SM scheme. Then, in Section 3, the performance analysis of the STBC-VBLAST system with TPA and the STBC-VBLAST-SM system is presented. Simulation results and performance comparisons are given in Section 4. Finally, concluding remarks are drawn in Section 5.

*Notation:* We use bold lowercase and capital letters to denote or column vectors and matrices, respectively. $\left(\begin{array}{c} n\\ k \end{array}\right)$ denotes the binomial coefficient. ${\left(.\right)}^{*}$ and ${\left(.\right)}^{H}$ denote complex conjugation and Hermitian transposition, respectively. P(·) denotes the probability of an event. We use ⌊x⌋ and ${\left\lfloor x\right\rfloor }_{2^{p}}$, respectively, for the largest integer less than or equal to *x* and the largest integer less than or equal to *x*—that is, an integer power of two. $I_{N}$ denotes the N×N identity matrix.

## 2. System Model

### 2.1. STBC-VBLAST Transmitter with TPA

**Figure 1:**
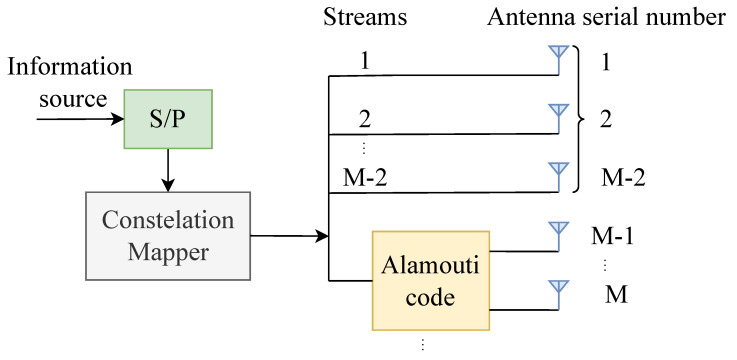
Block diagram for the STBC-VBLAST transmitter with TPA.

The block diagram of an STBC-VBLAST system with TPA having *M* transmit antennas and *N* receive antennas is shown in Figure 1. The information symbol sequence is demultiplexed into M−1 independent sub-streams. Sub-stream M−1 goes through Alamouti encoder and is transmitted on two antennas, while other sub-steams are transmitted on the other M−2 antennas independently. When the signals are transmitted over a quasi-static Rayleigh flat fading MIMO channel, the received N×2 signal matrix can be expressed as y=HPx+n, which can be also written as

(1) $$\left[\begin{array}{cc} y_{1,1} & y_{1,2}\\ y_{2,1} & y_{2,2}\\ \vdots & \vdots \\ y_{N,1} & y_{N,2} \end{array}\right]=HP\left[\begin{array}{cc} v_{1,1} & v_{1,2}\\ \vdots & \vdots \\ v_{M-2,1} & v_{M-2,2}\\ s_{M-1} & -s_{M}^{*}\\ s_{M} & s_{M-1}^{*} \end{array}\right]+n$$

where the element $v_{i,j}\left(i=1,\ldots ,M-2,j=1,2\right)$ indicates the *j*th symbol in the *i*th VBLAST layer and $s_{M},s_{M-1},-s_{M}^{*},s_{M-1}^{*}$ denote the symbols of STBC layers. H denotes the N×M channel matrix, and the entries of H are assumed to be i.i.d. complex Gaussian random variables with zero means and unit variances. n denotes the N×2 noise matrix, and the entries of n are assumed to be i.i.d. complex Gaussian random variables with variances $\sigma_{n}^{2}$.

The matrix

(2) $$P\;=\;\left[\begin{array}{cccccc} \sqrt{\frac{M-2\alpha }{M-2}} & 0 & \cdots & 0 & 0 & 0\\ 0 & \sqrt{\frac{M-2\alpha }{M-2}} & \cdots & 0 & 0 & 0\\ \vdots & \vdots & \ddots & \vdots & \vdots \\ 0 & 0 & \cdots & \sqrt{\frac{M-2\alpha }{M-2}} & 0 & 0\\ 0 & 0 & \cdots & 0 & \sqrt{\alpha } & 0\\ 0 & 0 & \cdots & 0 & 0 & \sqrt{\alpha } \end{array}\right]$$

decides the power of different layers. In traditional STBC-VBLAST systems, $P=I_{N}$ so that average energy of each transmitted symbol is the same. Therefore, to make full use of the power resource at the transmitter, we allocate the transmitter power of STBC-VBLAST systems. In STBC-VBLAST with TPA systems, the energy variable α is optimized to enhance the BER performance.

In an STBC-VBLAST system with TPA, when the modulation order is $2^{S}$, m=2S(M−1) bits can be transmitted in two slots. Therefore, the spectral efficiency of the STBC-VBLAST systems with TPA will be $k=\frac{1}{2}S\left(M-1\right)\left[\mathrm{bit}/s/\mathrm{Hz}\right]$.

### 2.2. MMSE and ZF Decoder for STBC-VBLAST Systems with TPA

The signals transmitted from the *M* antennas use Rayleigh fading channels and interfere with each other at the receiver. The received matrix over two time slots in (1) can be rearranged into a single vector as $y_{\mathrm{eff}}=H_{\mathrm{eff}}P_{\mathrm{eff}}x_{\mathrm{eff}}+W_{\mathrm{eff}}$ or (4). That is, the matrix $P_{\mathrm{eff}}$ can be expressed as

(3) $$P_{\mathrm{eff}}\;=\;\left[\begin{array}{cccccc} \sqrt{\frac{M-2\alpha }{M-2}} & 0 & \cdots & 0 & 0 & 0\\ 0 & \sqrt{\frac{M-2\alpha }{M-2}} & \cdots & 0 & 0 & 0\\ \vdots & \vdots & \ddots & \vdots & \vdots \\ 0 & 0 & \cdots & \sqrt{\frac{M-2\alpha }{M-2}} & 0 & 0\\ 0 & 0 & \cdots & 0 & \sqrt{\alpha } & 0\\ 0 & 0 & \cdots & 0 & 0 & \sqrt{\alpha } \end{array}\right]$$

It is worth noting that the dimension of $P_{\mathrm{eff}}$ is (2M−2)×(2M−2), which is M×M for P. The obtained system is equivalent to a spatial multiplexing scheme; thus, we can decode the transmitted symbols with the well-known ZF and MMSE decoder.

(4) $$\left[\begin{array}{c} y_{1,1}\\ y_{2,1}\\ \vdots \\ y_{N,1}\\ y_{1,2}^{*}\\ y_{2,2}^{*}\\ \vdots \\ y_{N,2}^{*} \end{array}\right]=\left[\begin{array}{cccccccc} h_{1,1} & \cdots & h_{1,M-2} & 0 & \cdots & 0 & h_{1,M-1} & h_{1,M}\\ h_{2,1} & \cdots & h_{2,M-2} & 0 & \cdots & 0 & h_{2,M-1} & h_{2,M}\\ \vdots & \cdots & \vdots & \vdots & \cdots & \vdots & \vdots & \vdots \\ h_{N,1} & \cdots & h_{N,M-2} & 0 & \cdots & 0 & h_{N,M-1} & h_{N,M}\\ 0 & \cdots & 0 & h_{1,1}^{*} & \cdots & h_{1,M-2}^{*} & h_{1,M}^{*} & -h_{1,M-1}^{*}\\ 0 & \cdots & 0 & h_{2,1}^{*} & \cdots & h_{2,M-2}^{*} & h_{2,M}^{*} & -h_{2,M-1}^{*}\\ \vdots & \cdots & \vdots & \vdots & \cdots & \vdots & \vdots & \vdots \\ 0 & \cdots & 0 & h_{N,1}^{*} & \cdots & h_{N,M-2}^{*} & h_{N,M}^{*} & -h_{N,M-1}^{*} \end{array}\right]P_{\mathrm{eff}}\left[\begin{array}{c} v_{1,1}\\ \vdots \\ v_{M-2,1}\\ v_{1,2}\\ \vdots \\ v_{M-2,2}\\ s_{M-1}\\ s_{M} \end{array}\right]+\left[\begin{array}{c} z_{1,1}\\ z_{2,1}\\ \vdots \\ z_{N,1}\\ z_{1,2}^{*}\\ z_{2,2}^{*}\\ \vdots \\ z_{N,2}^{*} \end{array}\right]$$

Inspired by [23,24], the ZF and MMSE of the STBC-VBLAST-TPA receiver architectures are proposed. When the channel matrix is assumed to be estimated perfectly at the receiver and $\tilde{H}=H_{\mathrm{eff}}P_{\mathrm{eff}}$, the ZF decoded symbol vector ${\hat{x}}_{\mathrm{ZF}}$ and the MMSE decoded symbol vector ${\hat{x}}_{\mathrm{MMSE}}$ are given by

(5) $${\hat{x}}_{\mathrm{ZF}}=W_{\mathrm{ZF}}*y,$$

(6) $${\hat{x}}_{\mathrm{MMSE}}=W_{\mathrm{MMSE}}*y$$

where

(7) $$W_{\mathrm{ZF}}={\left({\tilde{H}}^{H}\tilde{H}\right)}^{-1}{\tilde{H}}^{H},$$

(8) $$W_{\mathrm{MMSE}}={\left[{\tilde{H}}^{H}\tilde{H}+\sigma_{n}^{2}I_{2(M-1)}\right]}^{-1}{\tilde{H}}^{H}.$$

According to [22], the number of complex arithmetic operations, including complex multiplications and additions, is used to measure complexity. In general, the total complex arithmetic operations required of MMSE STBC-VBLAST without TPA number $8{\left(M-1\right)}^{3}+4\left(8N-1\right){\left(M-1\right)}^{2}+4N\left(M-1\right)$. As $P_{\mathrm{eff}}$ is a diagonal matrix, the process of $\tilde{H}=H_{\mathrm{eff}}P_{\mathrm{eff}}$ requires $M^{2}$ complex multiplications, so that the MMSE decoder of STBC-VBLAST with TPA consumes $8{\left(M-1\right)}^{3}+4\left(8N-1\right){\left(M-1\right)}^{2}+4N\left(M-1\right)+M^{2}$ complex arithmetic operations. Meanwhile, the total complex arithmetic operations of ZF STBC-VBLAST without TPA number $8(M-1{)}^{3}+4(8N-1)(M-1{)}^{2}+2(2N-1)(M-1)$, and for ZF STBC-VBLAST with TPA, the total complex arithmetic operations number $8(M-1{)}^{3}+4\left(8N-1\right)(M-1{)}^{2}+2\left(2N-1\right)\left(M-1\right)+M^{2}$. Obviously, the process of TPA influences computational complexity slightly.

### 2.3. STBC-VBLAST-SM Transmitter

**Figure 2:**
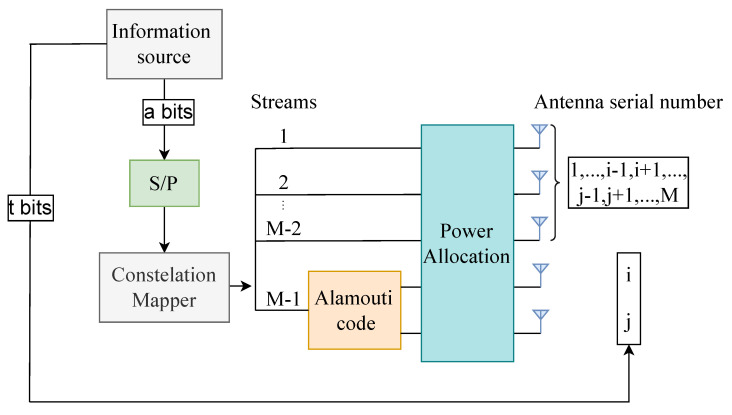
Block diagram for the STBC-VBLAST-SM transmitter.

By allocating power for different transmit antennas, the constellation sets of STBC and VBLAST layers are divided, which inspired us to utilize the SM scheme for higher spectral efficiency. For convenience, we call the improved scheme STBC-VBLAST-SM. The block diagram of an STBC-VBLAST-SM system with *M* transmit and *N* receive antennas is shown in Figure 2, in which Alamouti’s STBC is transmitted from the *i*th antenna and *j*th antenna and VBLAST symbols are transmitted on other antennas. The source bits are divided into a bits and k bits, carrying the information of APM symbols and antenna indices, respectively.

The received N×2 signal matrix can be expressed as y=HERPx+n, rewritten as

(9) $$\left[\begin{array}{cc} y_{1,1} & y_{1,2}\\ y_{2,1} & y_{2,2}\\ \vdots & \vdots \\ y_{N,1} & y_{N,2} \end{array}\right]=HERP\left[\begin{array}{cc} v_{1,1} & v_{1,2}\\ \vdots & \vdots \\ v_{M-2,1} & v_{M-2,2}\\ s_{M-1} & -s_{M}^{*}\\ s_{M} & s_{M-1}^{*} \end{array}\right]+n$$

where the diagonal matrix P represents the transmit power, as for STBC-VBLAST-TPA. $E=E_{i,M-1}E_{j,M},i\neq j$ denotes the permutation matrix that swaps the *i*th row and the *j*th row for the (M−1)th row and the *M*th row.

(10) $$R=\left[\begin{array}{cccccc} 1 & 0 & \cdots & 0 & 0 & 0\\ 0 & 1 & \cdots & 0 & 0 & 0\\ \vdots & \vdots & \ddots & \vdots & \vdots & \vdots \\ 0 & 0 & \cdots & 1 & 0 & 0\\ 0 & 0 & \cdots & 0 & exp(j\theta ) & 0\\ 0 & 0 & \cdots & 0 & 0 & exp(j\theta ) \end{array}\right]$$

denotes the matrix that rotates the constellation sets of STBC layers and $\theta =\frac{\pi }{2}$ for the BPSK modulation mode and θ=0 for the other modulation mode. In Section 4, the simulation results show the significance of rotation in detail.

Simultaneously, when modulation order is $2^{S}$, the number of possible antenna combinations for the transmission of STBC symbols is $c={\left\lfloor \left(\begin{array}{c} M\\ 2 \end{array}\right)\right\rfloor }_{2^{p}}$, where *p* is a positive integer, resulting in that l=p+2S(M−1) bits can be transmitted in two slots. Consequently, the spectral efficiency of such an STBC-VBLAST-SM system will be $g=\frac{1}{2}p+S\left(M-1\right)\left[\mathrm{bit}/s/\mathrm{Hz}\right]$, which is $\frac{1}{2}p\left[\mathrm{bit}/s/\mathrm{Hz}\right]$ more than the corresponding traditional STBC-VBLAST system and STBC-VBLAST-TPA system.

In the following, an algorithm to design the STBC-VBLAST-SM scheme is given.

**Step 1:** Calculate the number of possible antenna combinations for the transmission of Alamouti’s STBC according to the total number of transmit antennas *M*, i.e., $\left(\begin{array}{c} M\\ 2 \end{array}\right)$, from which, pick $c={\left\lfloor \left(\begin{array}{c} M\\ 2 \end{array}\right)\right\rfloor }_{2^{p}}$ possibilities to generate *c* types of permutation matrix $E_{q},q=1,...,c$. Divide the information source into “t bits” and “a bits,” which are mapped as SM symbols and APM symbols, respectively; the proportions of “t bits” and “a bits” are $\frac{p}{p+2\left(M-1\right)\left(\log_{2}S\right)}$ and $\frac{2\left(M-1\right)\left(\log_{2}S\right)}{p+2\left(M-1\right)\left(\log_{2}S\right)}$.

**Step 2:** Determine the rotation matrix R according to the modulation order $2^{S}$. Get the proper matrix P that controls the average power by simulating the theoretical BER performance in Section 3 in a certain application.

**Step 3:** Construct the codebook containing *l* codewords by mapping a block of information bits into two information carrying units:

- The VBLAST and STBC symbols, chosen from a complex signal constellation diagram, i.e., the transmission matrix x.
- The corresponding E, chosen from the set of $E_{q},q=1,...,c$

and multiplying x with P, R and E.

### 2.4. ML Decoder for the STBC-VBLAST-SM and STBC-VBLAST-TPA Systems

In this subsection, we formulate the ML decoder for the STBC-VBLAST-SM and STBC-VBLAST with TPA schemes. As the STBC-VBLAST-SM system with *M* transmit and *N* receive antennas is considered in the presence of a quasi-static Rayleigh flat fading MIMO channel, the received N×2 matrix is given by

(11) $$y=HX_{\chi }+n$$

where $X_{\chi },\chi =1,2,...,2^{l}$ are the N×2 transmission matrices and $E\left\{\mathrm{tr}\left(X_{\chi }^{H}X_{\chi }\right)\right\}=2$. The optimal ML decoder can search over all possible cases of $X_{\chi }$ to find the matrix in favor of minimizing the following metric:

(12) $${\hat{X}}_{\chi }=\arg\min_{X_{\chi },\chi =1,2,...,2^{l}}{∥y-HX_{\chi }∥}^{2}.$$

Similarly to the STBC-VBLAST-SM scheme, the corresponding STBC-VBLAST system with TPA can be represented by

(13) $$y=HX_{\Phi }+n$$

where $X_{\Phi },\Phi =1,2,...,2^{m}$ are the N×2 transmission matrices and $E\left\{\mathrm{tr}\left(X_{\Phi }^{H}X_{\Phi }\right)\right\}=2$. The optimal ML decoder is given by

(14) $${\hat{X}}_{\Phi }=\arg\min_{X_{\Phi },\Phi =1,2,...,2^{m}}{∥y-HX_{\Phi }∥}^{2}.$$

## 3. Performance Analysis

In this section, we analyze the error performances of STBC-VBLAST with TPA and STBC-VBLAST-SM. To begin with, in STBC-VBLAST-SM systems, *l* bits are transmitted during two consecutive symbol intervals. Furthermore, there are $2^{l}$ different transmission matrices denoted by $X_{1},X_{2},\ldots ,X_{2^{l}}$ here for convenience. An upper bound on the average bit error probability (BEP) is given by the well-known union bound [25]:

(15) $$P_{b}\leq \frac{1}{2^{l}}\sum_{i=1}^{2^{l}}\sum_{j=1}^{2^{l}}\frac{P\left(X_{i}\to X_{j}\right)n_{i,j}}{l}$$

where $P\left(X_{i}\to X_{j}\right)$ is the pairwise error probability (PEP) of the deciding matrix $X_{j}$, given that the matrix $X_{i}$ is transmitted, and $n_{i,j}$ is the number of bits in error between the matrices $X_{j}$ and $X_{i}$. The conditional PEP of the STBC-VBLAST system is calculated as

(16) $$P\left(X_{i}\to X_{j}|H\right)=Q\left(\sqrt{\frac{\rho }{2}}∥\left(X_{j}-X_{i}\right)H∥\right)$$

where $Q\left(x\right)=\left(1/\sqrt{2\pi }\right)\int_{x}^{\infty }e^{-y^{2}/2}dy$ and ρ indicates the average SNR at each receive antenna. Average over the channel matrix H, and then the unconditional PEP is obtained by using the moment generating function (MGF) [25] approach:

(17) $$P\left(X_{i}\to X_{j}\right)=\frac{1}{\pi }\int_{0}^{\pi /2}{\left(\frac{1}{1+\frac{\rho \lambda_{i,j,1}}{4\sin^{2}\phi }}\right)}^{n_{R}}{\left(\frac{1}{1+\frac{\rho \lambda_{i,j,2}}{4\sin^{2}\phi }}\right)}^{n_{R}}d\phi$$

where $\lambda_{i,j,1}$ and $\lambda_{i,j,2}$ are the eigenvalues of the distance matrix of the distance matrix $\left(X_{i}-X_{j}\right){\left(X_{i}-X_{j}\right)}^{H}$.

Consequently, the union of the BER of STBC-VBLAST-SM systems can be expressed as

(18) $$P_{b}\leq \frac{1}{2^{l}}\sum_{i=1}^{2^{l}}\sum_{j=1}^{2^{l}}\frac{\frac{n_{i,j}}{\pi }\int_{0}^{\pi /2}{\left(\frac{1}{1+\frac{\rho \lambda_{i,j,1}}{4\sin^{2}\phi }}\right)}^{n_{R}}{\left(\frac{1}{1+\frac{\rho \lambda_{i,j,2}}{4\sin^{2}\phi }}\right)}^{n_{R}}d\phi }{l}.$$

As *m* bits are transmitted during two consecutive symbol intervals in STBC-VBLAST systems with TPA, which are denoted by $X_{1},X_{2},\ldots ,X_{2^{m}}$, an upper bound on the average BEP is given by.

As the transmission matrices can be regarded as a kind of space time coding, the derivation of theoretical error performance of STBC-VBLAST with TPA is similar to that of STBC-VBLAST-SM. When *m* bits are transmitted during two consecutive symbol intervals in an STBC-VBLAST-SM system, the BER is expressed as

(19) $$P_{b}\leq \frac{1}{2^{m}}\sum_{i=1}^{2^{m}}\sum_{j=1}^{2^{m}}\frac{\frac{n_{i,j}}{\pi }\int_{0}^{\pi /2}{\left(\frac{1}{1+\frac{\rho \lambda_{i,j,1}}{4\sin^{2}\phi }}\right)}^{n_{R}}{\left(\frac{1}{1+\frac{\rho \lambda_{i,j,2}}{4\sin^{2}\phi }}\right)}^{n_{R}}d\phi }{m}.$$

The BER of STBC-VBLAST-SM and STBC-VBLAST-TPA are evaluated in the next section with different system parameters.

## 4. Simulation Results

In this section, we present a range of numerical BER performance simulation results of the STBC-VBLAST; STBC-VBLAST with TPA, termed STBC-VBLAST-TPA; and STBC-VBLAST-SM with different numbers of transmit antennas and receive antennas and make comparisons. In all simulations, a Rayleigh quasi-static fading channel was assumed to be known (or estimated perfectly) at the receiver. We use (M,N,S) notation to represent an MIMO configuration with *M* transmit and *N* receive antennas, and $2^{S}$-ary phase shift keying.

**Figure 3:**
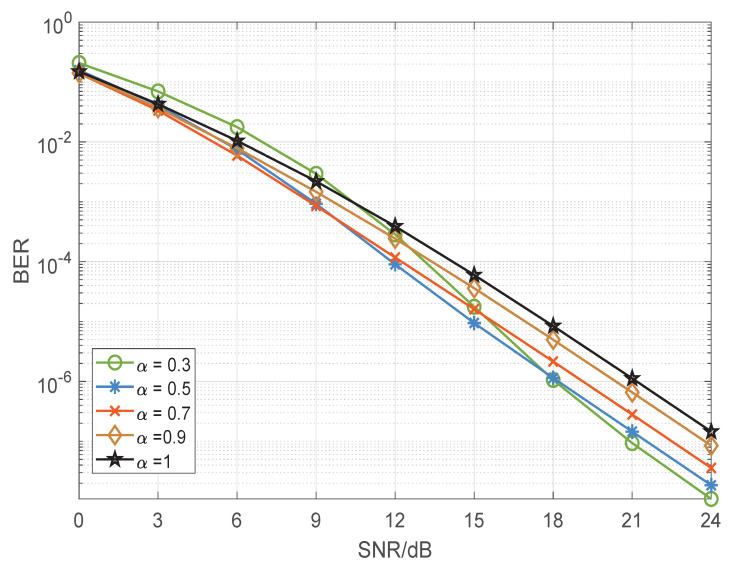
BER performance of the binary phase shift keying-modulated STBC-VBLAST-TPA system of α=0.3,0.5,0.7,0.9,1 having M=3 and N=3.

**Figure 4:**
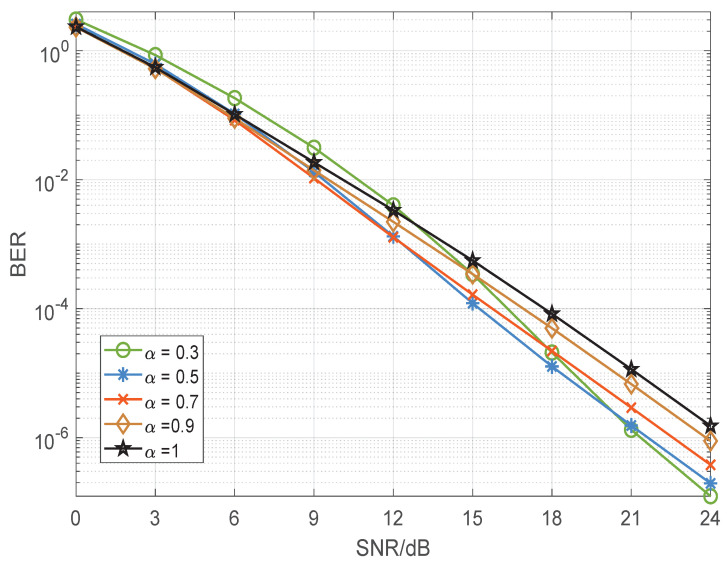
BER performance of the quadrature phase shift keying modulated STBC-VBLAST-TPA system of α=0.3,0.5,0.7,0.9,1 having M=3 and N=3.

In order to accurately illustrate the simulation results, we need to make it clear that when α=1, the traditional STBC-VBLAST and STBC-VBLAST-TPA are equivalent. The theoretical BER performances of BPSK and quadrature phase shift keying (QPSK)-modulated STBC-VBLAST-TPA systems with α=0.3,0.5,0.7,0.9,1 having M=3 and N=3 are shown in Figure 3 and Figure 4, from which we can make the following observation. To begin with, the STBC-VBLAST-TPA scheme obviously improves the BER performance compared to the traditional STBC-VBLAST scheme, though with slightly increased complexity. Secondly, the optimal α varies with modulation mode, MIMO configuration and SNR. As a result, when we design an STBC-VBLAST-TPA system, it is necessary to choose suitable α according to the theoretical performance shown in Section 3 and certain application requirements.

**Figure 5:**
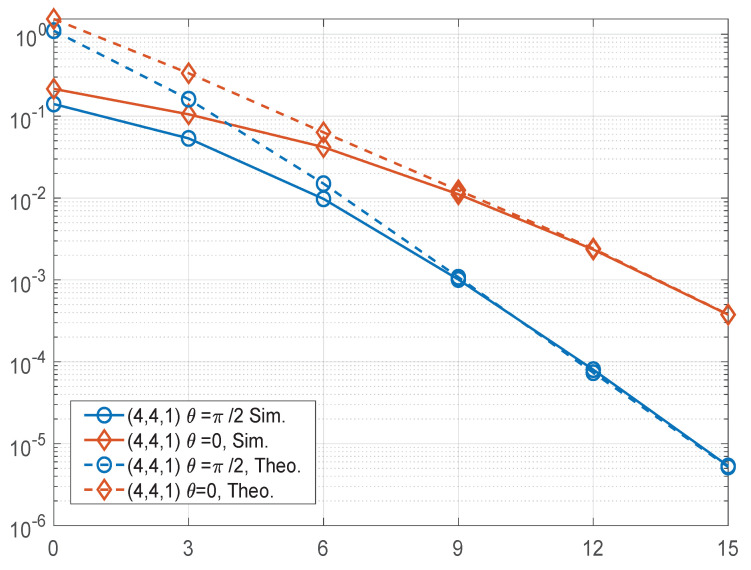
Performance comparisons of the (4, 4, 1) STBC-VBLAST-SM systems having $\theta =\frac{\pi }{2}$ and θ=0.

Figure 5 shows the theoretical upper bound and the BER performance curves of the BPSK modulated STBC-VBLAST-SM systems with θ=0 and $\theta =\frac{\pi }{2}$ having M=4 and N=4. The parameter α was set to 0.5. The figure verifies that for BPSK-modulated STBC-VBLAST-SM systems, the rotation of constellation sets of STBC layers can effectively improve the BER performance.

**Figure 6:**
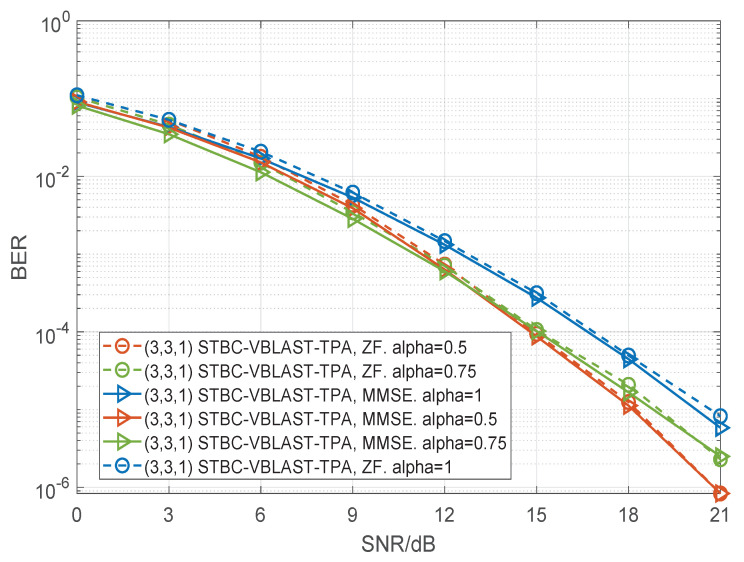
Performance comparisons of the (3,3,1) STBC-VBLAST-TPA system with α=1, α=0.5 and α=0.75, employing MMSE and ZF decoders.

**Figure 7:**
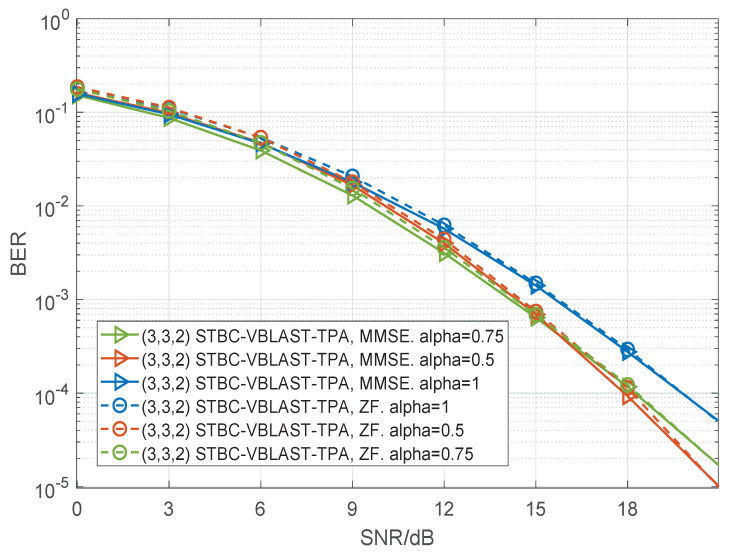
Performance comparisons of the (3,3,2) STBC-VBLAST-TPA system with α=1, α=0.5 and α=0.75, employing MMSE and ZF decoders.

**Figure 8:**
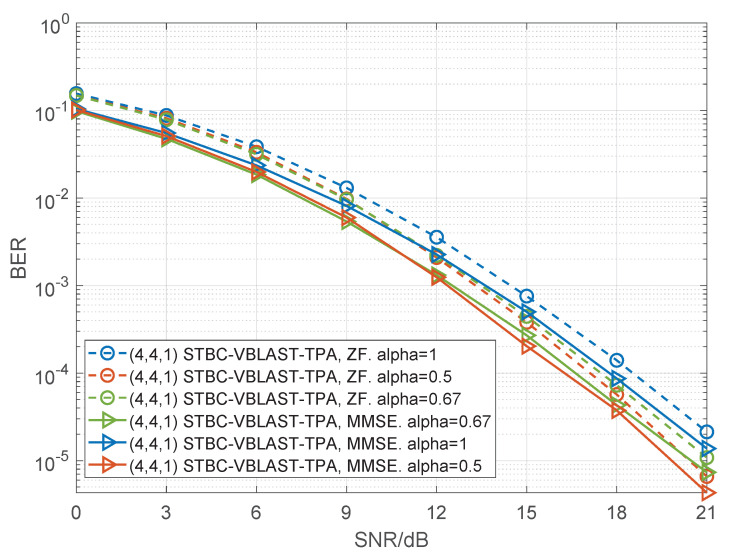
Performance comparisons of the (4,4,1) STBC-VBLAST-TPA system with α=1, α=0.5 and α=0.667, employing MMSE and ZF decoders.

In Figure 6, Figure 7 and Figure 8, we present the BER performance curves of the STBC-VBLAST-TPA scheme employing an MMSE decoder and a ZF decoder with different MIMO configurations. The parameter α was set to be 0.5 or 1, and $\frac{M}{2(M-1)}$, i.e., the average energy of each sub-stream was equal. More specifically, we assumed M=3 and α=0.75; M=4 and α=0.667. By these figures we can see that when SNR is high, STBC-VBLAST-TPA systems with α=0.5 show lower BER than those with α=1 and $\alpha =\frac{M}{2(M-1)}$, which means the STBC-VBLAST-TPA scheme can provide lower BER performance than the conventional STBC-VBLAST scheme.

**Figure 9:**
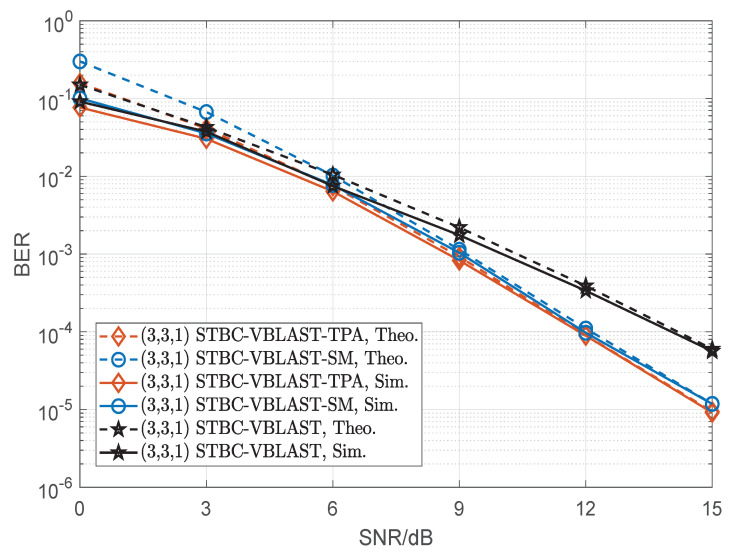
Performance comparisons of the (3,3,1) STBC-VBLAST-SM system, STBC-VBLAST-TPA system and traditional STBC-VBLAST system, where 2.5bits/s/Hz for the STBC-VBLAST-SM system and 2bits/s/Hz for the STBC-VBLAST-TPA and STBC-VBLAST systems.

**Figure 10:**
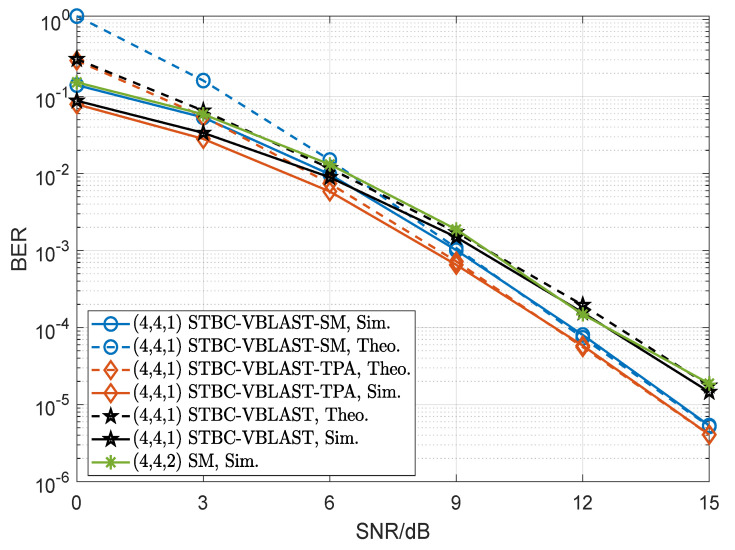
Performance comparisons of the (4,4,2) SM system, (4,4,1) STBC-VBLAST-SM system, STBC-VBLAST-TPA system and traditional STBC-VBLAST system, where 3bits/s/Hz for the STBC-VBLAST-SM system and 4bits/s/Hz for the STBC-VBLAST-TPA and STBC-VBLAST systems.

**Figure 11:**
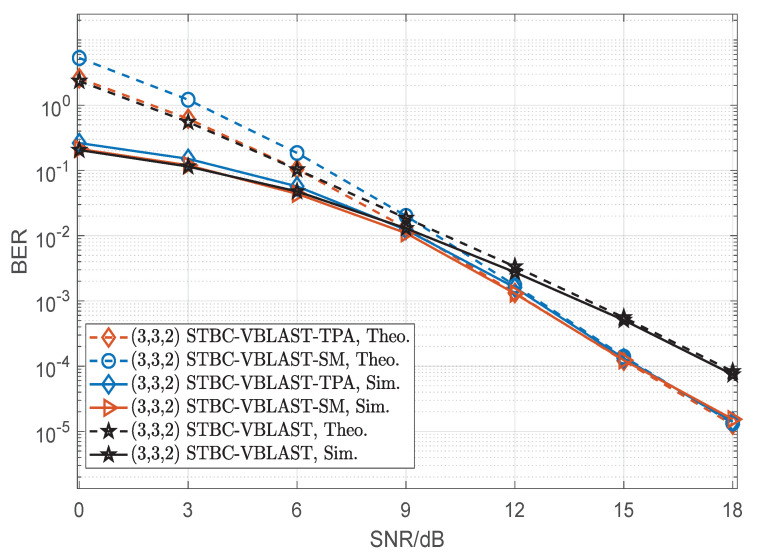
Performance comparisons of the (3,3,2) STBC-VBLAST-SM system, STBC-VBLAST-TPA system and traditional STBC-VBLAST system, where 4.5bits/s/Hz for the STBC-VBLAST-SM system and 4bits/s/Hz for the STBC-VBLAST-TPA and STBC-VBLAST systems.

Figure 9, Figure 10 and Figure 11 compare the BER performances of SM, STBC-VBLAST-SM, STBC-VBLAST-TPA and STBC-VBLAST having different MIMO configurations employing the ML decoder. Unless otherwise specified, the α of the STBC-VBLAST-SM and STBC-VBLAST-TPA systems was set to be 0.5. More explicitly, Figure 9 shows the theoretical and simulation performances of the (3,3,1) STBC-VBLAST-SM system at 2.5bits/s/Hz, and those of the (3,3,1) STBC-VBLAST-TPA and STBC-VBLAST systems at 2bits/s/Hz. Figure 10 presents the comparison of the (4,4,2) SM system at 4bits/s/Hz and the (4,4,1) STBC-VBLAST-SM system at 4bits/s/Hz with the (4,4,1) STBC-VBLAST-TPA and STBC-VBLAST systems at 3bits/s/Hz. Figure 11 compares the case of the (3,3,2) STBC-VBLAST-SM system at 4.5bits/s/Hz with the (3,3,2) STBC-VBLAST-TPA and STBC-VBLAST systems at 4bits/s/Hz. These figures provide the following observations. First of all, with ML decoders, STBC-VBLAST-SM and STBC-VBLAST-TPA offer higher diversity gains than traditional STBC-VBLAST and SM. Secondly, when M=N, STBC-VBLAST-SM systems can provide higher spectral efficiency than STBC-VBLAST-TPA and STBC-VBLAST systems with a little compromise in the BER performance. Finally, as the number of transmitter antennas grows with the spectral efficiency, the gap of BER performance between STBC-VBLAST-SM and STBC-VBLAST-TPA increases unavoidably.

## 5. Conclusions

In this paper, we mainly introduced two new transmission schemes, named STBC-VBLAST-TPA and STBC-VBLAST-SM. By allocating the transmit power, STBC-VBLAST-TPA is capable of improving the BER performance of STBC-VBLAST effectively. In STBC-VBLAST-SM systems, the antenna indices of Alamouti’s STBC are utilized to convey extra information, and as a result, spectral efficiency is improved. It has been shown via computer simulations and also supported by a theoretical upper-bound analysis that STBC-VBLAST-SM and STBC-VBLAST-TPA offer improvements compared with conventional STBC-VBLAST in various MIMO configurations.

## Data Availability

Not applicable.
